# Anti-Angiogenic Properties of Ginsenoside Rg3 Epimers: In Vitro Assessment of Single and Combination Treatments

**DOI:** 10.3390/cancers13092223

**Published:** 2021-05-06

**Authors:** Maryam Nakhjavani, Eric Smith, Kenny Yeo, Helen M. Palethorpe, Yoko Tomita, Tim J. Price, Amanda R. Townsend, Jennifer E. Hardingham

**Affiliations:** 1 Molecular Oncology, Basil Hetzel Institute, The Queen Elizabeth Hospital, Woodville South, SA 5011, Australia; maryam.nakhjavani@adelaide.edu.au (M.N.); a1811332@student.adelaide.edu.au (K.Y.); yoko.tomita@sa.gov.au (Y.T.); jennifer.hardingham@adelaide.edu.au (J.E.H.); 2 Adelaide Medical School, University of Adelaide, Adelaide, SA 5005, Australia; timothy.price@sa.gov.au (T.J.P.); amanda.townsend@sa.gov.au (A.R.T.); 3 Centre for Cancer Biology, University of South Australia and SA Pathology, Adelaide, SA 5000, Australia; helen.palethorpe@unisa.edu.au; 4 Oncology Unit, The Queen Elizabeth Hospital, Woodville South, SA 5011, Australia

**Keywords:** ginsenoside Rg3, response surface methodology, optimisation, epimer, angiogenesis

## Abstract

**Simple Summary:**

Angiogenesis is a critical step in tumour progression and metastasis. The application of current inhibitors of angiogenesis is accompanied by adverse effects. Therefore, there is a need for developing better treatments. *Panax ginseng* is a traditional herbal medicine that has been used by humans for thousands of years. 20(S) ginsenoside-Rg3 and 20(R) ginsenoside-Rg3 are two structurally similar molecules extracted from this plant, with distinct mechanisms of action. In this research, a combination of both of these molecules was optimised (C3) to inhibit angiogenesis, in lab settings. The results showed the role of C3 as a novel anti-angiogenic drug.

**Abstract:**

Tumour angiogenesis plays a key role in tumour growth and progression. The application of current anti-angiogenic drugs is accompanied by adverse effects and drug resistance. Therefore, finding safer effective treatments is needed. Ginsenoside Rg3 (Rg3) has two epimers, 20(S)-Rg3 (SRg3) and 20(R)-Rg3 (RRg3), with stereoselective activities. Using response surface methodology, we optimised a combination of these two epimers for the loop formation of human umbilical vein endothelial cell (HUVEC). The optimised combination (C3) was tested on HUVEC and two murine endothelial cell lines. C3 significantly inhibited the loop formation, migration, and proliferation of these cells, inducing apoptosis in HUVEC and cell cycle arrest in all of the cell lines tested. Using molecular docking and vascular endothelial growth factor (VEGF) bioassay, we showed that Rg3 has an allosteric modulatory effect on vascular endothelial growth factor receptor 2 (VEGFR2). C3 also decreased the VEGF expression in hypoxic conditions, decreased the expression of aquaporin 1 and affected AKT signaling. The proteins that were mostly affected after C3 treatment were those related to mammalian target of rapamycin (mTOR). Eukaryotic translation initiation factor 4E (eIF4E)-binding protein 1 (4E-BP1) was one of the important targets of C3, which was affected in both hypoxic and normoxic conditions. In conclusion, these results show the potential of C3 as a novel anti-angiogenic drug.

## 1. Introduction

Tumour angiogenesis is a critical step in tumour growth, survival, and metastasis. Several pro- and anti-angiogenic factors and signaling pathways contribute to regulate angiogenesis and facilitate tumour growth and metastasis [1,2,3]. The key driver of angiogenesis is the signaling of vascular endothelial growth factor receptor 2 (VEGFR2). VEGFR2 is activated upon interaction with its major ligand, VEGF. Hence, VEGF; VEGFR2; or the downstream signaling of VEGFR2, including PI3K/AKT, could be potential key targets in anti-angiogenesis drug development. Currently, the clinically approved anti-angiogenic agents are either antibodies against VEGF such as bevacizumab or small molecule tyrosine kinase inhibitors (TKIs). The administration of bevacizumab in advanced cancer patients could be accompanied by severe and sometimes fatal adverse effects, including hematological disorders, respiratory disorders, perforation and hemorrhage in the gastrointestinal system, and nervous system disorders [4]. TKIs also cause hematological and non-hematological events that may limit the application of treatment [5]. Furthermore, the administration of current anti-angiogenic treatments may also be limited because of drug resistance [6]. Therefore, developing effective less-toxic treatments is a fundamental effort for improving patient outcomes and it is the main aim of this research.

Epimers of ginsenoside Rg3 (Rg3), SRg3, and RRg3 are some of the most important pharmacologically active members of the ginsenosides family of chemicals extracted from *Panax ginseng* [7]. These molecules seem to be suitable anti-angiogenic candidates for drug development studies, because several studies have described their effects of inhibiting angiogenesis, and have shown their potential as anti-cancer agents (reviewed in [8,9]). Furthermore, in vitro and in vivo studies in animals and humans have shown tolerability and a low toxicity profile for these molecules (reviewed in [8,9]). These factors make Rg3 epimers intriguing candidates. In this regard, one important aspect of pharmacology of these epimers is their stereoselective anti-cancer action. We previously showed that these epimers have stereoselective activities for the inhibition of the migration and invasion of triple-negative breast cancer cell lines [10]. In addition, we showed that only SRg3 blocks the water transport function of aquaporin 1 (AQP1) [10], a protein that plays important roles in angiogenesis, tumour growth, and metastasis [11,12,13]. Furthermore, other studies have shown the stereoselectivity of these epimers on ion channels [14], the relaxation of the swine coronary artery [15], the anti-oxidant effect [16], promotion of immune system [17,18], and the inhibition of epithelial–mesenchymal transition [19]. Considering this stereoselective anti-cancer activity, these epimers should be considered as separate drugs that could be combined. 

For the first time, in this research, the concentrations of these epimers in combination was optimised to yield the highest anti-angiogenic efficacy. The optimal combination was determined using response surface methodology (RSM), a statistical and experiment design modelling process, which aims at reducing the number of experiments and costs associated with the experiment design [20]. In recent years, RSM has gained popularity in drug design [21], drug interaction [22], and combination therapy in cancer treatment studies [23]. It describes a three-dimensional dose–response surface, measures drug interactions, and defines the optimised combination of two drugs [24]. In this study, the efficacy of the optimal combination of Rg3 epimers was confirmed in migration and proliferation assays in human and murine endothelial cells. The mode of cell death and several potential intracellular targets of this combination that play roles in angiogenesis were studied. These targets included the expression of VEGF, activation of VEGFR2, signaling of AKT downstream of the activation of VEGFR2, and expression of AQP1. Because of the essential role of hypoxia in driving angiogenesis in a rapidly growing tumour, the role of this combination was studied in both normoxic and hypoxic conditions. [25].

## 2. Materials and Methods

### 2.1. Reagents, Cell Lines, and Cell Culture

Human umbilical vein endothelial cell (HUVEC) and its media, endothelial cell growth medium-2 (EBM-2; Clonetics, Lonza, Belgium), were purchased from Lonza, Belgium. Murine endothelial cell lines, 2H-11 and 3B-11, and human triple-negative breast cancer cell line MDA-MB-231 were purchased from the American Type Culture Collection (Manassas, VA, USA) and maintained in Dulbecco’s Modified Eagle Medium (DMEM; Life Technologies, Carlsbad, CA, USA), supplemented with 10% fetal bovine serum (Corning, Corning, NY, USA), 50 U/mL penicillin, and 50 µg/mL (Life Technologies). The cells were used within the first 10 passages. SRg3 (>98%) and RRg3 (>98%) (ChemFaces^®^, Wuhan, China) were dissolved in dimethyl sulfoxide (DMSO, HYBRI-MAX, Sigma-Aldrich, St. Louis, MO, USA). Aliquots of SRg3 and RRg3 at 6.5 and 12.7 mM, respectively, as the maximum concentrations of Rg3 epimers in aqueous media, were stored at −20 °C. The concentration of DMSO in the experiments did not exceed 0.8%, as described previously [10].

### 2.2. Response Surface Methodology (RSM)

To develop the RSM, the central composite design technique was employed with three levels, namely: low, mid, and high values corresponding to −1, 0, and +1, respectively, for the input parameters. The input parameters were the concentration of SRg3 and RRg3, which ranged from 0–100 µM for SRg3 and 0–50 µM for RRg3. Table 1 represents the values corresponding to low, mid, and high bounds of concentrations for the Rg3 epimers. The design matrix used in the RSM analysis is shown in Supplementary Table S1. To optimise the combination of concentrations, the RSM model reduced the total experiments to 13 iterations, with loop formation being the “main measurable target parameter”.

Following optimising the combination, two other combinations were used to confirm the validity of the RSM model. These two combinations (C1 and C2) are as follows, which were tested along with the optimised combination (C3). Combination 1 (C1): SRg3 (12.5 µM) + RRg3 (6.25 µM). Combination 2 (C2): SRg3 (25 µM) + RRg3 (12.5 µM).

### 2.3. Proliferation Assay

A crystal violet assay was performed as previously described [26]. Briefly, cells were seeded at 800 cells per well of a 96-well plate and were cultured overnight. Single or combination concentrations of Rg3 epimers were added to the wells, and the absorbance was read at 595 nm at 3 time points, on days 0, 1, and 3, in order to assess the effect of the Rg3 epimers on the proliferation of the endothelial cell lines. The experiment included six replicates and the data are shown as mean ± standard deviation (SD).

### 2.4. Flow Cytometric Analysis of Cell Death

The cells were seeded at 5 × 10^4^ cells per well on six-well plates overnight and were then exposed to Rg3 combinations for three days. Then, the samples were collected and stained using the Annexin-V-FLUOS staining kit (Roche Diagnostics, Mannheim, Germany), as previously described [10]. The samples were analysed in the BD FACSCanto II (BD Biosciences, San Jose, CA, USA) and FlowJo software, v 10.4 (FlowJo, LLC, Ashland, OR, USA). The experiment was performed in triplicate and the data are shown as mean ± SD.

### 2.5. Flow Cytometric Analysis of Cell Cycles

The cells were seeded at 5 × 10^4^ cells per well on six-well plates, cultured overnight, and then exposed to C3 for 3 days. The cells were collected, fixed, stained, and analysed using BD FACSCanto II and FlowJo software, v10.4, as previously described [10]. The experiment was performed in triplicate and the data are shown as mean ± SD.

### 2.6. Migration Assay

A migration assay was performed based on the previously described method [27]. Briefly, HUVECs, 2H-11, and 3B-11 cells, either not pretreated or pretreated for 3 days with Rg3 epimers, were seeded in 96-well plates at 3.5 × 10^4^, 1.2 × 10^4^, and 4 × 10^4^ cells per well, respectively, and were incubated overnight. A circular scratch was made in the cell monolayer. The area of the circular wound was measured based on a time of 0 and 10 h (murine endothelial cells), or 16 h (HUVEC), using ImageJ software (version 1.53a, National Health of Institute, Bethesda, MD, USA). The experiment included six replicates per treatment and the data are shown as mean ± SD.

### 2.7. Loop Formation Assay

A loop formation assay was optimised based on the cell proliferation index, viability, and cell number, and was performed as previously described [28]. Endothelial cells were seeded at 1.5 × 10^4^ cells per well of a µ-plate (Ibidi, Martinsried, Germany) coated with Matrigel^®^ (Corning) according to the manufacturer’s protocol. The number of loops formed was counted at 16 h for HUVEC and 4 h for 2H-11 and 3B-11. The results are presented relative to the vehicle control. The experiment was performed in triplicate and the data are shown as mean ± SD.

### 2.8. Molecular Docking

For the molecular docking of Rg3 on the VEGF receptors, the SMILES structures of Rg3, sorafenib, and lenvatinib were obtained from PubChem. The crystal structure of VEGFR2 (2XIR and 3V2A) and VEGFR1 (5EX3) were from the protein data bank of NCBI (RCSB PDB). The UCSF Chimera program (version 1.15-mac64) and Autodock Vina algorithm (version 1-1-1-mac-catalina-64bit) were used to build the 3D structure of Rg3 and perform the molecular docking. The prediction of the Gibbs free energy of the protein-ligand binding was based on the flexible ligand docking simulations run within the docking grids on the interaction site of each protein, as previously described [10].

### 2.9. VEGFR2 Specific Interaction

To study the interaction between Rg3 epimers and VEGFR2, a VEGF bioassay kit (Promega, Madison, WI, USA) was used. It is a bioluminescent assay using KDR/NFAT-RE HEK293 cells. Upon activation of VEGFR2, intracellular signals triggered NFAT-RE-mediated luminescence. The experiment was performed according to the manufacturer’s protocol. Briefly, the cells were seeded in white, flat-bottom 96-well assay plates (Delta Surface ^TM^, Thermo Scientific, Roskilde, Denmark). Serial dilutions of SRg3 and RRg3 at final maximum concentrations of 100 and 50 µM were used alone or in combination with VEGF-A (recombinant VEGF, Promega, Madison, WI, USA) at a constant final concentration of 35 ng/mL (80% effective concentration). Bevacizumab (Avastin^®^, a maximum final concentration of 6 µg/mL) and VEGF-A (a maximum final concentration of 0.1 µg/mL) were used as the controls. The cells were incubated with the drugs for 6 h before a 10 min incubation with the Bio-Glo™ Reagent. Bioluminescence was measured using a FLUOstar Optima microplate reader (BMG LABTECH, Offenburg, Germany). The relative luminescence units (RLU) in each well were subtracted from the background. The experiment was performed in duplicate. GraphPad Prism (version 9.0.0 for Mac, GraphPad Software, San Diego, CA, USA, www.graphpad.com (accessed on 11 March 2021)) was used for plotting the dose–response curves (non-linear regression using log(inhibitor) vs. normalised response) and calculating the half inhibitory concentration (IC_50_).

### 2.10. Quantitative PCR for the Expression of AQP1

The cells were seeded at 0.5 × 10^5^ cells per well on six-well plates and were incubated overnight. Then, the cells were treated with Rg3 for 3 days at a normoxic (21% O_2_) or hypoxic (0.1% O_2_) condition. PureLink RNA mini kit (Life Technologies) was used to extract RNA and 20 ng RNA was used for reverse transcription using iScript cDNA Synthesis Kit (Bio-Rad Laboratories, Hercules, CA, USA). The duplex TaqMan Gene Expression Assays for aquaporin-1 (AQP1; Hs01028916_m1; Life Technologies) and the reference gene CCSER2 (HS00982799_mH, Life Technologies) was used in the study. Three biological replicates were used. Reactions were performed in triplicate and were analysed as previously described [10].

### 2.11. Enzyme-Linked Immunosorbent Assay (ELISA) for the Expression of VEGF-A

HUVEC and MDA-MB-231 cells were seeded on six-well plates at 1 × 10^5^ cells per well on a 96-well plate. After overnight culture, the cells were exposed to C3 for three days. The expression of VEGF in these cells was compared in normoxic and hypoxic conditions. Following treatment, the supernatants were collected and centrifuged to pellet any debris. The cells were then lysed with a RIPA Lysis and Extraction Buffer (Pierce Biotechnology, Rockford, IL, USA) and the total protein was measured using Bio-Rad protein assay (Bio-Rad Laboratories). VEGF production was measured using the human VEGF-A ELISA Kit (RayBiotech, Norcross, GA, USA). The experiment was performed in duplicate, and the results are shown as mean ± SD.

### 2.12. Western Blotting for the Expression of Proteins Involved in Migration and Invasion

The total cell lysates were prepared and quantified as described above. Western blot was performed as previously described [28]. The anti-aquaporin-1 antibody [EPR20325] (ab219055, Abcam, Cambridge, UK, 1:1000) and goat anti-rabbit IgG H&L (ab6721, Abcam, 1:3000) were used as the primary and secondary antibodies, respectively. The experiments were repeated three times and the results are shown as mean ± SD.

### 2.13. AKT Pathway Phosphorylation Array

To assess the effect of Rg3 on the signaling of AKT, a Human/Mouse AKT Pathway Phosphorylation Array C1 (RayBiotech) was used. The HUVEC cells were pretreated with Rg3 or a vehicle (DMSO) for three days at normoxic and hypoxic conditions, and then the protein was collected using lysis buffer, protein inhibitor, and phosphatase inhibitor, as per the manufacturer’s protocol. The protein concentration was determined using Bio-Rad Protein Assay Dye Reagent Concentrate (Bio-Rad Laboratories, Hercules, CA, USA). The density of each dot was measured using Image Lab™ Software (version 6.1). The results are shown as the mean ± SD of the two replicates.

### 2.14. Statistical Analysis

The results were analysed using parametric one-way or two-way analysis of variance using GraphPad Prism (version 9.0.0 for mac, GraphPad Software, San Diego, CA, USA, www.graphpad.com). The results are presented as mean ± SD for two to eight replicates, with *p* < 0.05.

## 3. Results

### 3.1. Optimisation of Concentration Combination of SRg3 and RRg3

**Figure 1:**
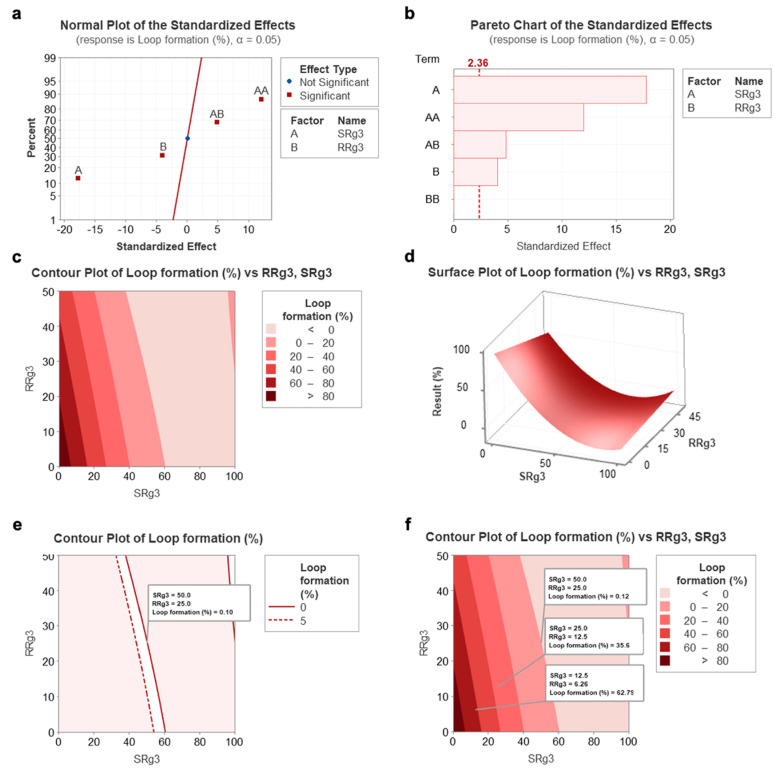
The calculated results of the response surface methodology developed using the central composite design technique for the optimisation of SRg3 and RRg3 drugs. (**a**) Standardised effect chart showing the critical parameters that need to be investigated, (**b**) Pareto chart analysis for loop formation data that reflects the effectiveness of each parameter, (**c**) contour plot, (**d**) surface plot for the percentage of loop formation following a combination of SRg3 or RRg3, (**e**) contour plot highlighting the area with the best efficacy for the combination, and (**f**) contour plot showing the predicted responses of two other combination treatments. A and B stand for SRg3 and RRg3, respectively, and AB represents the combination of SRg3 and RRg3. AA and BB show a mathematical expression of high concentrations of SRg3 and RRg3, respectively.

The results of the response surface methodology modelling are depicted in Figure 1. Parameters A (SRg3), B (RRg3), and the combination of both (AB), all have significant effects (Figure 1a). Notably, AA is defined as a high concentration of SRg3, which is included as a reference. For further analysis, the Pareto chart analysis for the loop formation data (Figure 1b) reflects the effectiveness of each parameter and shows the critical parameters that needed to be investigated in this study. This chart shows that the concentration of SRg3 (A), RRg3 (B), and the combination of both drugs (AB) are key parameters playing a major role in the anti-angiogenic effects. The highest effect is sourced from SRg3, followed by the combination of both drugs and RRg3. Accordingly, the key parameter requiring optimisation is the combination of both drugs (AB), which shows a plausible efficacy and reduces the concentration needed of each if used singly. Both the Pareto analysis and standardised effect plots showed that the combination parameter is a key factor determining the efficacy of loop formation. By optimising the concentrations, the optimum region for a concentration of both drugs to give the minimum loop formation was identified, and is shown in a contour plot (Figure 1c) and surface plot (Figure 1d).

Notably, AA is defined as a high concentration of SRg3, which is included as a reference. For further analysis, the Pareto chart analysis for the loop formation data (Figure 1b) reflects the effectiveness of each parameter and shows the critical parameters that need to be investigated in this study. This chart shows that the concentration of SRg3 (A), RRg3 (B), and the combination of both drugs (AB) are key parameters playing a major role in the anti-angiogenic effects. The highest effect is sourced from SRg3, followed by the combination of both drugs and then RRg3. Accordingly, the key parameter requiring optimisation is the combination of both drugs (AB), which shows a plausible efficacy and reduces the concentration needed of each if used singly. Both the Pareto analysis and standardised effect plots showed that the combination parameter is a key factor determining the efficacy of loop formation. By optimising the concentrations, the optimum region for a concentration of both drugs to give the minimum loop formation was identified and is shown in a contour plot (Figure 1c) and surface plot (Figure 1d).

**Figure 2:**
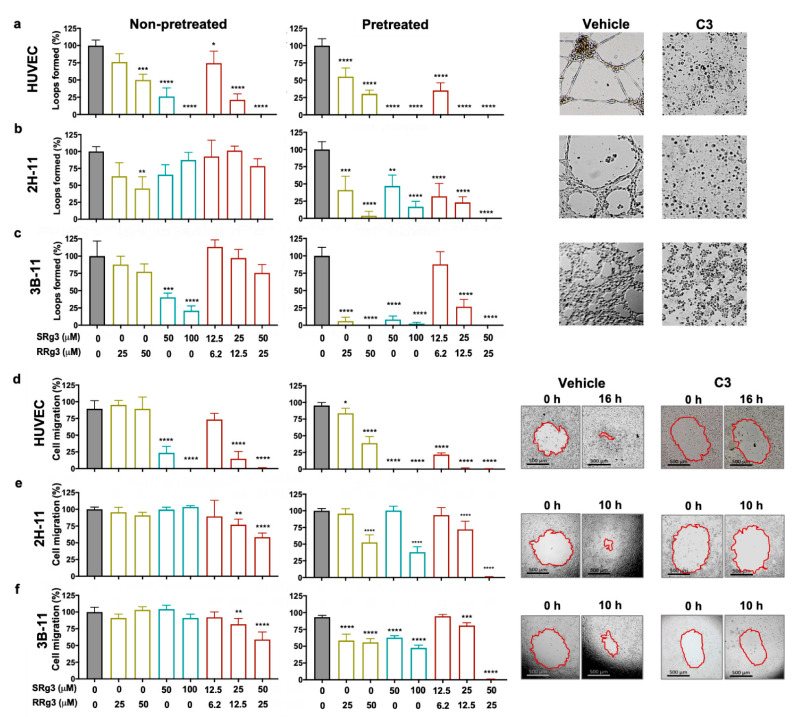
Effect of Rg3 epimers on loop formation (**a**–**c**) and migration (**d**–**f**) of human umbilical vein endothelial cell (HUVEC), 2H-11, and 3B-11 cells. Analysis of the loop formation and migration was performed at two timepoints; non-pretreated cells and 3-day pre-treated cells. Treatments are shown on the x axis, and (**a**–**c**) show the results of the loop formation in the HUVEC, 2H-11, and 3B-11 cell lines, respectively, at peak loop formation timepoints—16 h for HUVEC and 4 h for 2H-11 and 3B-11 cells. The experiments were done in triplicate and the results are presented as mean ± standard deviation (SD; *p* < 0.05). (**d**–**f**) show the results of cell migration in HUVEC, 2H-11 and 3B-11 cell lines, respectively. Results are presented as mean ± SD of 3 and 6 replicates for loop formation and migration assays, respectively (*p* < 0.05). The images represent the pre-treated cells. C3 represents a combination of 50 µM SRg3 + 25 µM RRg3. * *p* < 0.05, ** *p* < 0.01, *** *p* < 0.001 and **** *p* < 0.0001.

Accordingly, different areas, shown with different colours (Figure 1c), show the percentage of loop formation in response to the combination of concentrations of SRg3 and RRg3. As represented in Figure 1e, by narrowing down the identified region of the result to 0–5% loop formation, the response of the combination of 50 µM SRg3 + 25 µM RRg3 (C3) was minimised to 0.1%, in which loop formation was almost completely suppressed. Notably, a concentration of 50 µM SRg3 is a concentration that blocks AQP1 water channels [10], which, in combination with 25 µM RRg3, gives a minimum loop formation. To validate the results of this RSM model, two other combinations (C1 and C2) were considered and tested for loop formation. Figure 2a shows the results of the validation of the RSM model on HUVEC cells. As shown in Figure 1f, C1 and C2 are predicted to provide responses of 60–80% and 20–40% loop formation, respectively. In Figure 2a it is shown that C1 and C2 give mean responses of 74% and 22%, which is within the predicted regions in Figure 1f. Therefore, the identified concentration of C3 was used to conduct the rest of the experiments.

### 3.2. Effect of Rg3 on Loop Formation and Migration of Endothelial Cells

To show the effects of Rg3 epimers alone and in combination, loop formation and migration assays were performed at two-time points: on non-pretreated cells and three-day pretreated cells (Figure 2). In the non-pretreated state, HUVECs were the most sensitive of the three cell types to inhibitory effects of single and combination of Rg3 epimers, with C3 being the most effective combination to completely inhibit loop formation (Figure 2a–c) and cell migration (Figure 2d–f). In these cells, in this state, a dose–response relationship was observed for a single or combination of Rg3 epimers. The loop formation with RRg3 at 25 µM and 50 µM (*p* = 0.0001) was 76% and 50%, respectively. Loop formation with 50 µM SRg3 was inhibited by 74% (*p* < 0.0001) and was completely inhibited with 100 µM SRg3 (*p* < 0.0001). 

With combinations of C1 and C2, loop formation was reduced to 74% (*p* = 0.0348) and 21% (*p* < 0.0001), while C3 completely inhibited loop formation (*p* < 0.0001). In this state, the murine 2H-11 and 3B-11 cell lines were less sensitive to the inhibitory effects of Rg3 and the treatment required more time to show an inhibitory action in these cell lines. 2H-11 was more sensitive to the effects of RRg3, and at 25 µM and 50 µM, loop formation was 63% and 45% (*p* = 0.0023), respectively (Figure 2b). However, only SRg3 inhibited loop formation in 3B-11. With 50 µM and 100 µM SRg3, 40% (*p* = 0.0005) and 21% (*p* < 0.0001) loop formation occurred, respectively (Figure 2c). Although the combinations did not significantly inhibit the loop formation of murine endothelial cell lines in the non-pretreated state, a dose–response pattern was observed with these treatments. 

To study the time-dependency of the effects of Rg3, a three-day pretreatment was performed. Following this pretreatment of cells with Rg3, the inhibitory effects of treatment were exacerbated in all of the tested cells. In HUVEC, RRg3 at 25 and 50 µM inhibited loop formation by 35 and 70%, respectively (*p* < 0.0001), and RRg3 completely inhibited loop formation (*p* < 0.0001). With C1, only 35% loop formation occurred, and no loops formed with C2 and C3 (*p* < 0.0001). In the pretreated state, the effect of single and combination drugs increased in both murine cell lines. In 2H-11, 25 and 50 µM RRg3 decreased loop formation by an average of 59% (*p* = 0.0004) and 96% (*p* < 0.0001), respectively. SRg3 at 50 and 100 µM inhibited loop by 53% (*p* < 0.0026) and 83% (*p* < 0.0001), respectively. C1, C2 and C3, inhibited loop formation by 68%, 78% and 100% (*p* < 0.0001), respectively. In pretreated 3B-11 cells, all single drugs inhibited loop formation by more than 90% (*p* < 0.0001), and C2 and C3 inhibited it by 73 and 100% (*p* < 0.0001), respectively. These results showed that Rg3 has a time- and dose-dependent effect on the inhibition of loop formation for endothelial cells. 

In the migration assay, the efficacy of single or a combination of Rg3 epimers was studied in non-pretreated or 3-day pretreated cells (Figure 2d–f). The trend of cells’ response in this assay was similar to the results of loop formation assay. Similar to the inhibitory effects of Rg3 on loop formation, HUVEC was the most sensitive cell to the anti-migration effects of Rg3 (Figure 2d). 

In non-pretreated HUVEC, only SRg3 inhibited cell migration by 66% and 80% for 50 and 100 µM, respectively (*p* < 0.0001; Figure 2d). C1, C2, and C3 inhibited loop formation dose-dependently by 16%, 75% (*p* < 0.0001), and 89% (*p* < 0.0001), respectively (Figure 2d). 2H-11 and 3B-11 were not sensitive to single epimers, but the C2 (*p* < 0.001) and C3 (*p*<0.0001) significantly inhibited cell migration. A three-day exposure of the cells with the drugs increased the effects of single drugs. In HUVEC, the inhibitory effects of RRg3 increased and both concentrations of SRg3 completely inhibited migration (*p* < 0.0001). In 2H-11, the inhibitory effects of higher concentrations of SRg3 and RRg3 increased, while in 3B-11, the effects of all single epimers were increased. In both cell lines, C3 almost completely inhibited cell migration (Figure 2e,f). This experiment also showed time- and dose-dependent inhibition of migration by Rg3 epimers and confirmed the results of Rg3 in the loop formation assay.

### 3.3. Anti-Proliferative Effects of Rg3 in Endothelial Cells

**Figure 3:**
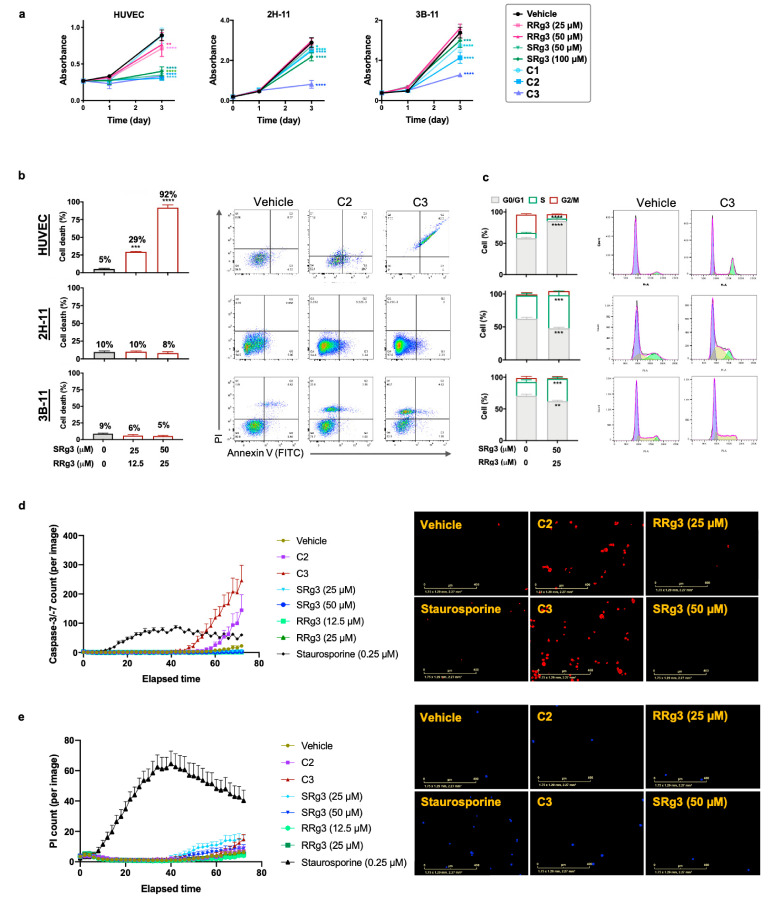
HUVEC, 2H-11, and 3B-11 cells were exposed to 0.8% dimethyl sulfoxide (DMSO) as a vehicle control or at concentrations of 25 and 50 µM RRg3; 50 and 100 µM SRg3; or three combinations of RRg3 + SRg3 at 6.2 + 12.5 (C1), 12.5 + 25 (C2), and 25 + 50 µM (C3). (**a**) The effect of single or combination Rg3 epimers on the proliferation of these cells in a three-day time frame. Each data point represents mean ± SD of six replicates. (**b**) The flow cytometric analysis of the induction of cell death and (**c**) cell cycle arrest in these cells by C2 and C3. Each data point represents mean ± SD of three replicates. (**d**) Activation of caspase 3/7, shown by red spots and (**e**) propidium iodide (PI) staining of cells shown by blue spots in HUVEC cells. Images are at 72 h and scale bars show 400 µm. Each data point represents mean ± SD of eight replicates. Statistical analyses were performed between the Rg3 and vehicle-treated cells (*p* < 0.05). * *p* < 0.05, ** *p* < 0.01, *** *p* < 0.001 and **** *p* < 0.0001.

As shown in Figure 3a, HUVEC was the most sensitive cell type to the anti-proliferative effects of Rg3. At equimolar concentrations, RRg3 was a less potent inhibitor of cell proliferation in HUVEC, while SRg3, C2, and C3 almost completely inhibited cell proliferation (*p* < 0.0001), and C1 had no significant inhibitory action. 2H-11 and 3B-11 were more sensitive to the combination of SRg3 and RRg3 compared with single epimers. In these two cell lines, C3 was the most effective inhibitor of cell proliferation (Figure 3a).

The induction of cell death was studied by staining the cells with annexin V and propidium iodide (PI; Figure 3b). In HUVEC cells, C2 and C3 induced about 29% (*p* = 0.0003) and 92% (*p* < 0.0001), respectively, cell death after three days of treatment. The cell death induced by C3 was associated with G0/G1 arrest in HUVEC (*p* < 0.0001; Figure 3c). Further studies showed that C2 and C3 induced the activation of caspase 3/7 in HUVEC by 536% and 980%, respectively (Figure 3d), with subsequent increases in the number of PI-positive cells (Figure 3e), consistent with late apoptosis. Interestingly, single epimers of Rg3 did not induce caspase activation in HUVEC. This shows that SRg3 and RRg3 in C2 and C3 combinations play a synergistic role in the induction of apoptosis in this cell. 

In contrast, in murine 2H-11 and 3B-11 endothelial cells, C2 and C3 did not induce significant cell death (Figure 3b), while the cells were arrested in S phase (Figure 3c). Induction of cell cycle arrest in the S-phase was by 42% (*p* = 0.0006) and 63% (*p* = 0.0001) in 2H-11 and 3B-11, respectively. Therefore, it seems that the major mechanism of the inhibition of proliferation in these two cell lines is via the induction of cell cycle arrest.

### 3.4. The Effect of Rg3 on VEGF, VEGFR2, and Their Interaction

**Figure 4:**
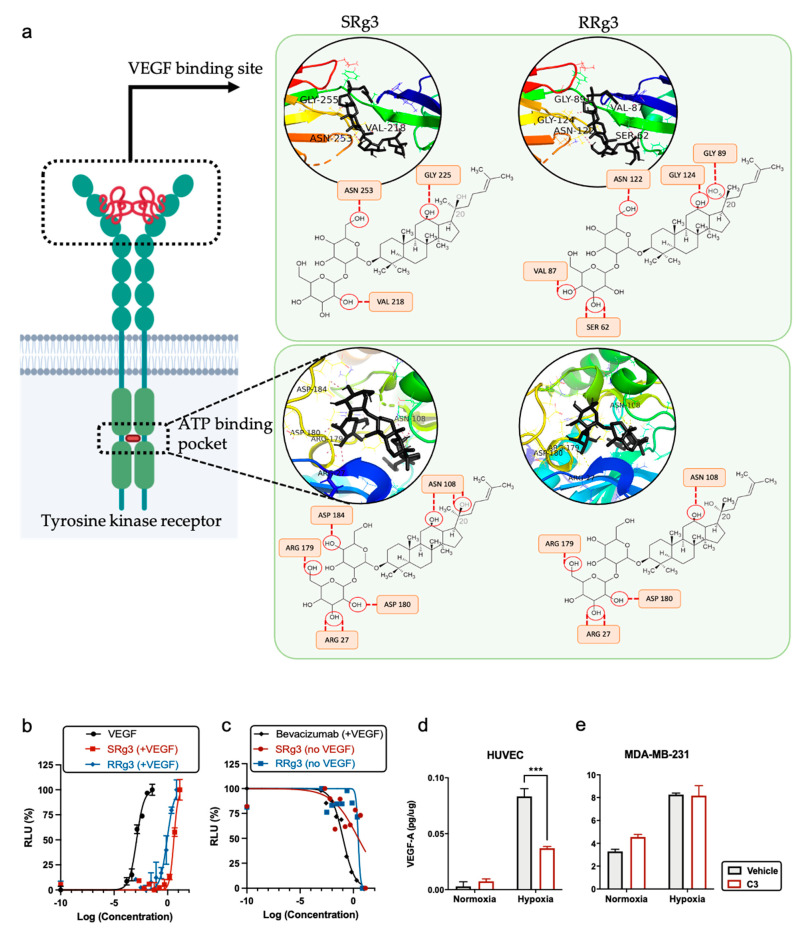
(**a**) A demonstration of the interaction between SRg3 and RRg3 (in black) with VEGFR2 at VEGF binding site or ATP-binding pocket. The interaction sites were predicted using molecular docking performed by AutoDock Vina algorithm. The predicted H-bonds between Rg3 and amino acid residues are shown with dashed lines. Dose–response curve of (**b**) VEGF, SRg3, and RRg3 in the presence of 35 ng/mL VEGF (stimulatory dose–response state) and (**c**) bevacizumab in the presence of 35 ng/mL VEGF, SRg3, and RRg3 alone (inhibitory dose–response state). Expression of VEGF in the presence of Rg3 in normoxic or hypoxic conditions in (**d**) HUVEC and (**e**) MDA-MB-231. The experiment was performed in duplicate, and the results are shown as mean ± SD, with *p* < 0.05. RLU—relative light units. *** *p* < 0.001.

To further investigate the role of Rg3 epimers in angiogenesis, molecular docking was performed on two of the receptors VEGF—VEGFR1 and VEGFR2. As a comparable reference, molecular docking was also performed on two small-molecule TKIs—sorafenib and lenvatinib—which are known to interact with and inhibit VEGFR activity [29]. Human VEGFR2 includes an extracellular site with seven immunoglobulin (Ig)-like domains, and an intracellular tyrosine kinase domain, which are connected with a short transmembrane and a juxta-membrane domain (Figure 4a) [30]. The results of the molecular docking between Rg3 epimers and VEGFR1 and VEGFR2 predicted good binding scores between TKIs and the ATP-binding pocket of these receptors (Table 2). Molecular docking of SRg3 and RRg3 at this site of VEGFR2 predicted that both epimers have a strong binding with this site of the receptor, with scores of −9.0 and −8.9 kJ/mol, respectively. These scores are comparable with the binding scores of sorafenib (−9.9 kJ/mol) and lenvatinib (−9.1 kJ/mol; Table 2). 

As shown in Figure 4a and summarised in Table 2, 8 and 5 H-bonds were predicted between VEGFR2 and the two epimers, SRg3 and RRg3, respectively. In both cases, Asn108, Asp180, Arg27, and Arg179 were suggested as potential H-bond residues. Although the ATP-binding cassette of VEGFR2 plays an important role in the activation of the receptor, the VEGF binding site in the extracellular side of the receptor is a key interaction site between VEGF and VEGFR2 to facilitate the intercellular signal transduction. Out of the 7 Ig-like domains, the first three domains, especially 2 and 3, mediate VEGF binding [31]. We performed a molecular docking on domains 2 and 3 of VEGFR2 and each of the Rg3 epimers. The results of this in silico study predicted binding scores of −7.2 and −7.0 kJ/mol for SRg3 and RRg3, respectively. These scores, together with the number of H-bond interactions with the receptor, were 3 and 5 H-bonds for SRg3 and RRg3, respectively, indicating strong binding. For both epimers, glycine, asparagine, and valine were the predicted amino acid residues to make H-bonds with each epimer at different positions of the domains, and provide affinity positions for H-bonds (Figure 4a). 

To investigate the interaction between Rg3 and VEGFR2, in vitro, a VEGF bioassay was conducted. Figure 4b,c shows two dose–response curves of VEGF in a stimulatory state and a bevacizumab inhibitory state, respectively. VEGF, as the activator of the receptor, shows a stimulatory dose–response curve (Figure 4b) with a half effective concentration (EC_50_) of 0.001 ng/mL (Table 3). The anti-VEGF monoclonal antibody, bevacizumab, antagonised the action of VEGF (Figure 4c) with the IC_50_ of 0.11 µg/mL (Table 3). 

To test the activity of Rg3 on VEGFR2, the bioassay was performed in two states: (i) in the presence of high levels (EC_80_) of VEGF, which is a condition that encourages angiogenesis and highly activates VEGFR2, and (ii) in the absence of VEGF to test whether the molecules alone have any stimulatory or inhibitory effect on the receptor. In the presence of an EC_80_ value of VEGF (35 ng/mL), Rg3 epimers shifted the VEGF dose–response curve to the right. This means that Rg3 epimers reduced the efficacy of VEGF for the activation of VEGFR2. The EC_50_ values of SRg3 and RRg3 in this state were about 28 and 6.5 µM (Table 3). This means that RRg3 is almost four-fold more potent than SRg3 at reducing the efficacy of VEGF. SRg3, although less potent, more effectively shifted the VEGF dose–response curve to the right (Figure 4b). 

To test whether the Rg3 epimers had any stimulatory effect on VEGFR2, the dose response curve was studied in the absence of VEGF. In this state, Rg3 epimers showed an almost steady response, except for the highest concentration (Figure 4c), and the regression analysis approach was not a plausible technique to fit a non-linear sigmoidal dose–response function, which resulted in poor R squared values (Table 3). However, the overall trend of their effect was inhibitory, as at the highest concentrations used (100 and 50 µM for SRg3 and RRg3, respectively), the response was minimised (Figure 4c). The decreased bioluminescence detected at the highest tested concentrations could be due to the cytotoxicity of the molecules, rather than the inhibitory effect of the drugs on the receptor.

**Figure 5:**
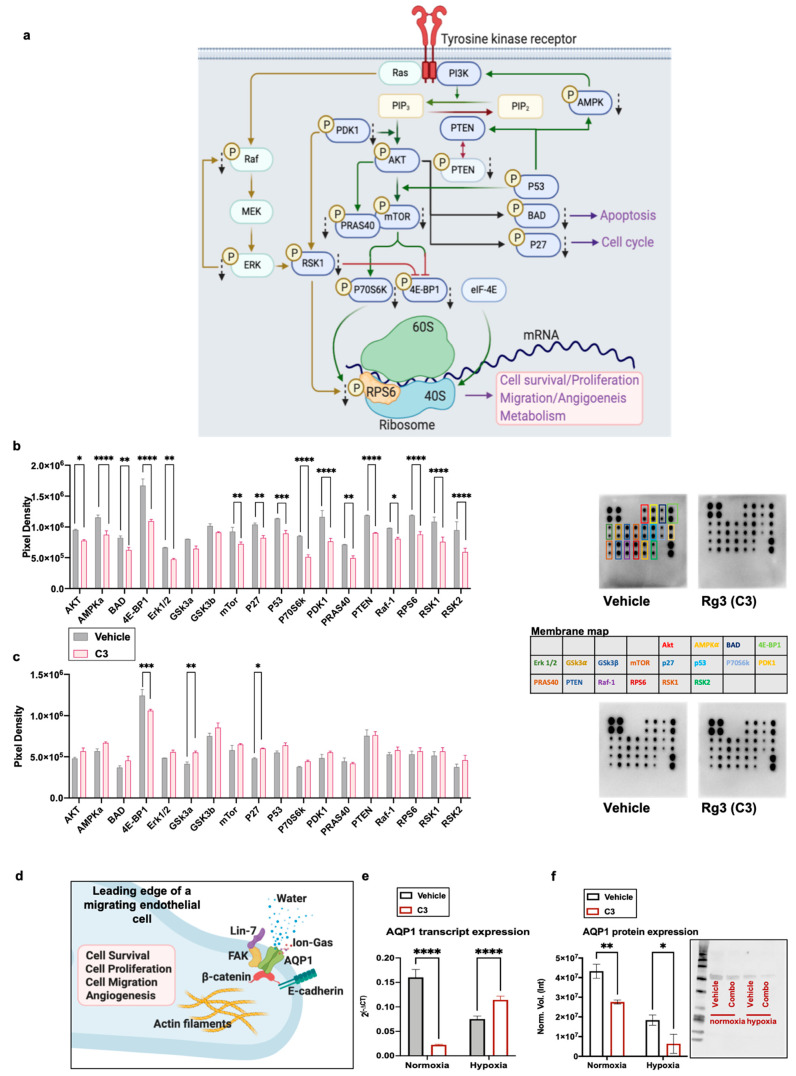
(**a**) A schematic diagram of the signaling of the downstream activation of a receptor tyrosine kinase, leading to cell survival, proliferation, migration, angiogenesis, and metabolism (created with BioRender.com (accessed on 11 March 2021)). The effects of C3 (50 µM SRg3 + 25 µM RRg3) on the phosphorylation of signaling proteins in PI3K/AKT signaling in HUVEC cells grown in (**b**) normoxia and (**c**) hypoxia (1% O_2_) conditions. The cells were exposed with C3 for three days. The data show the mean ± SD of two replicates with *p* < 0.05. (**d**) A schematic diagram of the role of AQP1 localised in the leading edge of an endothelial cell facilitating cell migration (reviewed in [9]) (created with BioRender.com). The cells were exposed with C3 for thee days in normoxic or hypoxic (1% O_2_) conditions. Expression levels of (**e**) AQP1 transcripts measured with QT-PCR or (**f**) proteins detected using the Western blotting technique in normoxic and hypoxic conditions are shown. The data represent the mean ± SD of three replicates with *p* < 0.05. * *p* < 0.05, ** *p* < 0.01, *** *p* < 0.001 and **** *p* < 0.0001.

Given the observed effects of Rg3 in this system and the results obtained from the molecular docking, it seems probable that Rg3 is an allosteric modulator of VEGFR2. In the absence of VEGF, as the primary ligand, SRg3 and RRg3 had a minimum activity on the receptor, while in the presence of VEGF, Rg3 epimers decreased the efficacy of VEGF-VEGFR2 interactions potentially by changing the conformation of the receptor. Furthermore, the efficacy of C3 on the VEGF expression in normoxic and hypoxic conditions in HUVEC and MDA-MB-231 cells was studied (Figure 4d,e). C3 did not have any significant effect on VEGF expression in MDA-MB-231 (Figure 5e), but in hypoxic HUVEC cells, when the expression of VEGF was significantly increased in vehicle-treated cells, Rg3 decreased this expression (*p* = 0.0008; Figure 4d).

### 3.5. Effect of Rg3 on the AKT Signalling Pathway and AQP1

Signaling of PI3K/AKT and its interaction with the Raf/MEK/ERK signal transduction pathway (Figure 5a) regulates several proteins controlling cell survival, proliferation, migration, and metabolism. To test whether C3 treatment has any effects on the signaling of AKT, a protein array was performed. C3 affected the phosphorylation of proteins downstream of the activation of AKT in both normoxia and hypoxia, although the effects in normoxia were more extensive (Figure 5). In normoxic conditions, Rg3 combined with C3 affected the phosphorylation of several proteins important in the signaling of AKT. The phosphorylation of AKT was decreased (*p* = 0.017) and regulators for the activation of AKT, including AMP-activated protein kinase (AMPK), phosphatase and tensin homolog (PTEN), and phosphoinositide-dependent kinase-1 (PDK1), were decreased (*p* < 0.0001) (Figure 5b). C3 also decreased the phosphorylation of the BCL2 associated agonist of cell death (BAD; *p* = 0.0048; Figure 5b), which plays important roles in AKT-mediated cell survival. C3 decreased the phosphorylation of cyclin-dependent kinase inhibitor 1B (p27^kip1^; *p* = 0.0015; Figure 5b), hence keeping it in its active form, which could cause p27^kip1^-mediated G1 arrest. With C3, the activation of p53 also decreased (*p* = 0.0003), which could also affect the activation of mTOR. 

This experiment showed that C3 decreased the phosphorylation of mTOR (*p* = 0.0045), PRAS40 (*p* = 0.0012), P70S6K (*p* < 0.0001), 4E-BP1 (*p* < 0.0001), and RPS6 (*p* < 0.0001). These proteins play roles in the translation function of migrating cells. Furthermore, C3 decreased the phosphorylation of Raf (*p* = 0.0187), ERK 1/2 (*p* = 0.0066), RSK1, and RSK2 (*p* < 0.0001). In hypoxic conditions, the C3 affected proteins included 4E-BP1 (*p* = 0.0003), glycogen synthase kinase-3a (GSK3a; *p* = 0.0086), and p27^kip1^ (*p* = 0.0323). 

AQP1 in combination with other proteins at the leading edge of a migrating cell facilitates cell migration (Figure 5d). We showed that in normoxic conditions, the levels of AQP1 transcript (*p* < 0.0001) and protein (*p* = 0.0268) were significantly decreased. In hypoxic conditions, although the transcript levels were increased, the protein levels were decreased (*p* = 0.0195; Figure 5e,f). In addition, in a mouse endothelial cell line, 3B-11, a significantly decreased expression of the AQP1 transcript was observed (Supplementary Figure S1). However, we did not detect any significant changes in the activation of focal adhesion kinase (FAK), as another player in the AQP1-facilitated migration in HUVECs (Supplementary Figure S2). Therefore, it seems that AQP1 is a more important protein in the C3-induced inhibition of migration in HUVECs. Furthermore, preliminary testing showed that in C2 treated HUVEC, the expression of the AQP1 transcript and protein and the activation of AKT were reduced (Supplementary Figure S3). 

## 4. Discussion

A few studies have investigated the mechanisms of action of Rg3 as an anti-angiogenic agent. Keung et al. (2016) showed that RRg3 exerted its anti-angiogenic effects via an increased expression of hsa-miR-520h, which targeted ephrin type-B receptor 2 (EphB2) and EphB4 as a mediator of cancer migration and angiogenesis [32]. In addition, it was shown that an unspecified epimer of Rg3 (64 µM) decreased the protein and transcript expression of VEGF, basic fibroblast growth factor (b-FGF), matrix metalloproteinase-2 (MMP-2), and MMP-9 [33]. Because of the stereoselective activity of Rg3 epimers, for the first time, in this study, C3 was introduced as an optimised combination of SRg3 and RRg3 and a novel anti-angiogenic agent. This combination showed time- and dose-dependent anti-angiogenic properties in vitro. HUVECs were more sensitive to these effects of Rg3 than to the murine endothelial cell lines. 

To further investigate the mechanisms involved in the anti-angiogenic properties of Rg3 and more specifically C3, (i) the effects of Rg3 on the VEGF–VEGFR2 interaction and (ii) the anti-angiogenic mechanisms Rg3 combination (C3) were studied. Molecular docking predicted good biding scores and VEGFR2, comparable to the binding of known TKIs. We showed that Rg3 has no stimulatory action on VEGFR2. The antiangiogenic effects observed by Rg3 are not comparable with a drug such as bevacizumab. Bevacizumab is a monoclonal antibody against VEGF, while Rg3 has several mechanisms, one of which is via the interaction with the activation of VEGFR2. For the first time, we showed that the interaction between Rg3 and VEGFR2 decreased the efficacy of VEGF on the system, working as an allosteric modulator. Allosteric modulators are of special interest in pharmacology. Since the introduction and successful treatment profile of benzodiazepines as allosteric ligands of γ-aminobutyric acid-A (GABA_A_) receptors, versus the toxic direct-acting agonists of this receptor, much more attention has been paid to finding and registering allosteric drugs for various diseases (reviewed in [34]). Allosteric modulators offer several advantages over orthosteric ligands, such as subtype selectivity within receptor families and less adverse side effects [34,35]. Of special interest are tyrosine kinases, which play roles in several human diseases such as cancer. The ATP-binding pocket of kinases is a highly conserved part, and this results in a low selectivity and, consequently, off-target and side effects for the inhibitors designed for this target. Other types of inhibitors either bind at the ATP site extending into an adjacent allosteric pocket, specifically bind to the allosteric pockets near the ATP pocket, or bind to allosteric sites more remote from the ATP pocket [34]. The fact that Rg3 has been administered to humans without any reported serious side effects (reviewed in [8,9]) could be evidence for the safety of Rg3 allosterism. This research provided evidence of the anti-VEGFR2 action of Rg3 epimers as one of the anti-angiogenic mechanisms of these molecules. To confirm the interaction site of Rg3 with VEGFR2, further experiments are required in future research. 

This research also provided evidence of the effectiveness of C3 in hypoxic conditions. This is especially important because of the importance of hypoxia in driving tumour invasiveness [36]. Hypoxia is a common feature of rapidly growing tumours, which affects tumour metabolism, metastasis, and resistance to chemotherapy [37], and is linked to a poor prognosis for several tumours (reviewed in [38]). Hypoxia leads to VEGF expression to encourage angiogenesis in the tumour. Increased VEGF expression promotes endothelial cell proliferation and migration, inhibits apoptosis in these cells, and facilitates the degradation of the extracellular matrix and endothelial cell migration and invasion [39]. Some solid tumours such as breast cancers overexpress VEGF and its receptors. This led to the development of anti-angiogenic drugs for these patients [40]. Our group is interested in developing novel treatments for breast cancer, in which VEGF expression is an independent prognostic factor and a possible target of treatment [41]. Both endothelial and breast cancer cells have an autocrine VEGF signaling pathway that supports angiogenesis and cancer progression [42]. Hence, we measured VEGF expression in both HUVEC and MDA-MB-231, in hypoxic and normoxic conditions, and showed that C3 significantly decreased VEGF expression only in hypoxic HUVEC cells and not in MDA-MB-231. Whether C3 has efficacy on other breast cancer cell lines should be further assessed. However, the responding cells, endothelial cells, play key roles in angiogenesis. 

Downstream of the activation of VEGFR2, several signaling pathways are activated, including PI3K/AKT/mTOR signaling, the activation of which is one of the hallmark signaling pathways in cancer and angiogenesis [43,44]. Therefore, inhibitors of mTOR signaling have gained plenty of attention in cancer treatment. Currently, 70 trials of the inhibitors of mTOR signaling are recruiting in several tumour types, such as breast, lung, colorectal, and hematological tumours (https://clinicaltrials.gov/ (accessed on 11 March 2021)). In addition to several cellular functions, the activation of mTOR also plays roles in VEGF production and angiogenesis [45]. Inhibitors of this pathway, inhibiting either PI3K/mTOR or mTOR alone, show anti-angiogenic properties (reviewed in [46]). For example, rapamycin, an inhibitor of mTORC1, also inhibits VEGF production and angiogenesis [47]. As reviewed before, in leukemic and ovarian cancer models, Rg3 affected PI3K/AKT signaling [8]. Therefore, we examined the effects of C3 on this signaling pathway in normoxic and hypoxic conditions. In the normoxic condition, except GSK3, all other tested proteins were affected with C3. The proteins that showed more than 30% decreased phosphorylation were those that were related to mTORC1 function, including PRAS40 (a component and substrate of mTORC1); P70S6K, which phosphorylates and activates RSP6, a component of 40S ribosomal subunit; and 4E-BP1, which, upon phosphorylation, releases eIF-4E, as one of the key components of ribosomal translation initiation for regulators of mTOR function including PDK1 and RSKs. PRAS40, when dephosphorylated, inhibits mTOR signaling, consequently decreasing ribosomal transcription via affecting the activation of 4E-BP1 and P70S6k, both of which play roles in tumour angiogenesis [48,49].

Decreased phosphorylation of 4E-BP1 was also observed both in hypoxic and normoxic HUVEC cells exposed to C3, thus implicating 4E-BP1 as having an important role in C3 mediated anti-angiogenic effects. In particular, as mTORC1, via mechanisms involving 4E-BP1, drives VEGF signaling in hypoxic conditions [50], it could be considered that the decreased expression of VEGF observed in these cells was mediated through mTORC1/4E-BP1. The leading edge of migrating cells is where many fundamental biological and biochemical processes occur to facilitate cell migration, including 4E-BP1, PRAS40, mRNAs, and translation initiation factors [51,52]. Therefore, one major mechanism of C3 could be via the inhibition of the translational function of mTOR. 

Additionally, hypoxia-induced-endothelial cell proliferation requires functional mTOR complexes [53]. C3, via the decreased phosphorylation of 4E-BP1, could decrease the functionality of mTORC1 and hence play a contributing role in the decreased proliferation of these cells. C3, in hypoxic conditions, caused minor increased levels of p-p27^Kip1^, which is a negative regulator of G1 cell cycle progression. It has been shown that GSK3 stabilises the levels of p27^Kip1^ and decreases cell proliferation [54] and hence, the observed minor increased levels of p27^Kip1^ could be a consequence of the deactivation of GSK3.

GSK3 is a constitutively active kinase, an activator of AKT, mTORC1, and mTORC2, which is in feedback and crosstalk with PI3K/AKT/mTOR. Phosphorylation on SER-21 (GSK3a) and SER-9 (GSK3b) is initiated by the growth factor activation of AKT/mTOR and inhibits GSK3 function [55], and C3 increased this phosphorylation to decrease the activation of this signaling. This could further decrease the functionality of mTORC1 and mTORC2. This is especially important, as mTORC2 is an essential cellular energy production element, which promotes cancer progression via lipid formation and fueling the PI3K/AKT/mTOR pathway [56]. It also plays roles in driving angiogenesis multiple myeloma, where mTORC2 inhibitors restrict angiogenesis in this tumour model [57]. mTORC2 is one of the molecular targets that is in advanced stages of translational application, and whether C3 has any inhibitory action on mTORC2 needs to be further investigated.

Some AQPs such as AQP1, AQP4, and AQP5 localise at the leading edge of migrating cells [58]. AQP1, for example, polarises at the leading edge, a phenomenon that is associated with an increased turnover of cell membrane protrusions and enhanced cell migration (reviewed in [12]). AQP1 was recognised as a pro-angiogenic factor [59], which, independent of VEGF, was required for the hypoxia-induced tube forming capacity of human retinal vascular endothelial cells [60]. AQP1-deficient cells were shown to have impaired migration and tube formation [61]. We have also shown that blockers of AQP1 impair angiogenesis [27,28]. In addition, using the molecular docking and oocyte swelling assay, we showed that Rg3 blocked AQP1 [10]. Therefore, it could be concluded that the blockage of AQP1 could contribute to the immediate inhibition of the loop formation observed. After a three-day pretreatment with C2 and C3, the protein expression of AQP1 was decreased. FAK, another important contributor to endothelial cell migration via VEGFR2-signalling or complexing with AQP1 (reviewed in [13]), did not seem to be involved in the mechanism of action of C3, which further highlights the role of AQP1 in this process.

## 5. Conclusions

In conclusion, we showed that Rg3 had a time- and dose-dependent inhibition of the migration and invasion of endothelial cells. The optimised combination of SRg3 and RRg3 inhibited the proliferation, migration, and invasion of endothelial cells. SRg3 and RRg3 potentiated each other’s action in activating caspase 3/7 and inducing apoptosis, which was the major anti-angiogenic mechanism. This action was measured after 3 days of exposure with the treatment. Besides the induction of apoptosis, other inhibitory mechanisms were also involved that assisted with the anti-angiogenic action of Rg3. As our studies showed, these molecules were allosteric modulators of VEGFR2, and therefore potentially had far fewer off-target effects with less clinical side effects expected. A reduced expression of VEGF and AQP1, and decreased PI3K/AKT/mTOR signaling are suggested mechanisms of this drug. Further studies are needed to confirm the anti-angiogenic effects of C3 in vivo.

## Figures and Tables

**Table 1:** Low, mid, and high values used for response surface methodology (RSM) model.

Parameter	Index	Concentration (µM)		
		Lowest value (−1)	Centre Value (0)	Highest Value (+1)
**SRg3**	A	0	50	100
**RRg3**	B	0	25	50

**Table 2:** Binding score (kJ/mol) of Rg3 epimers and growth factor receptors and the number of hydrogen binding (H-bond) predicted by Chimera program and Autodock vina algorithm.

Molecule	Binding Score (kJ/mol) (Number of H-Bonds)		
	VEGFR1	VEGFR2 ^1^	VEGFR2 ^2^
SRg3	4.8 (0)	−9.0 (8)	−7.2 (3)
RRg3	−7.4 (6)	−8.9 (5)	−7.0 (5)
Sorafenib	−4.9 (0)	−9.9 (0)	−
Lenvatinib	−8.9 (0)	−9.1 (0)	−

^1^ Interaction with ATP-binding pocket; ^2^ interaction with vascular endothelial growth factor (VEGF)-binding site.

**Table 3:** Calculated IC_50_ and EC_50_ values for VEGF (ng/mL), bevacizumab (µg/mL), SRg3 (µM), and RRg3 (µM) alone or in combination with 35 ng/mL VEGF, in interaction with VEGFR2. The experiment was performed in duplicate using the VEGF bioassay system (Promega) and was analysed using Prism software.

Compound	IC_50_	EC_50_	95% CI ^1^	R Squared
VEGF	−	0.001	0.001–0.002	0.9781
Bevacizumab	0.11	−	0.08–0.15	0.9644
SRg3	21.23	−	3.25–8008	0.3391
RRg3	20.67	−	15.06–30.82	0.6963
SRg3 + VEGF	−	27.95	23.79–32.44	0.9670
RRg3 + VEGF	−	6.52	4.84–8.66	0.9411

^1^ CI—confidence interval

## Data Availability

All of the data are available in the paper or as Supplementary Data.

## Supplementary Materials

The following are available online at https://www.mdpi.com/article/10.3390/cancers13092223/s1, Figure S1: Quantitative PCR for transcript expression of AQP1 in murine 3B-11 cell line following exposure with C3 for 3 days. Figure S2: Western blot analysis of activation of focal adhesion kinase (FAK) in HUVEC exposed to vehicle (V) or C3 (50 µM SRg3 + 25 µM RRg3), in normoxia or hypoxia conditions. Figure S3: HUVEC was exposed to vehicle (V) or C2 (25 µM SRg3 + 12.5 µM RRg3) for 3 days. Table S1: Design table developed for the RSM model.

## References

[1] Ollauri-Ibáñez C., Astigarraga I. (2021). Use of antiangiogenic therapies in pediatric solid tumors. Cancers.

[2] Maennling A.E., Tur M.K., Niebert M., Klockenbring T., Zeppernick F., Gattenlöhner S., Meinhold-Heerlein I., Hussain A.F. (2019). Molecular targeting therapy against EGFR family in breast cancer: Progress and future potentials. Cancers.

[3] He B., Ganss R. (2021). Modulation of the vascular-immune environment in metastatic cancer. Cancers.

[4] Taugourdeau-Raymond S., Rouby F., Default A., Jean-Pastor M.-J. (2012). Bevacizumab-induced serious side-effects: A review of the French pharmacovigilance database. Eur. J. Clin. Pharmacol..

[5] Hartmann J.T., Haap M., Kopp H.-G., Lipp H.-P. (2009). Tyrosine kinase inhibitors-a review on pharmacology, metabolism and side effects. Curr. Drug Metab..

[6] Haibe Y., Kreidieh M., El Hajj H., Khalifeh I., Mukherji D., Temraz S., Shamseddine A. (2020). Resistance mechanisms to anti-angiogenic therapies in cancer. Front. Oncol..

[7] Yun U.-J., Lee I.H., Lee J.-S., Shim J., Kim Y.-N. (2020). Ginsenoside Rp1, A ginsenoside derivative, augments anti-cancer effects of Actinomycin D via downregulation of an AKT-SIRT1 pathway. Cancers.

[8] Nakhjavani M., Hardingham J.E., Palethorpe H.M., Tomita Y., Smith E., Price T.J., Townsend A.R. (2019). Ginsenoside Rg3: Potential molecular targets and therapeutic indication in metastatic breast cancer. Medicines.

[9] Nakhjavani M., Smith E., Townsend A.R., Price T.J., Hardingham J.E. (2020). Anti-angiogenic properties of ginsenoside Rg3. Molecules.

[10] Nakhjavani M., Palethorpe H.M., Tomita Y., Smith E., Price T.J., Yool A.J., Pei J.V., Townsend A.R., Hardingham J.E. (2019). Stereoselective anti-cancer activities of ginsenoside Rg3 on triple negative breast cancer cell models. Pharmaceuticals.

[11] Nico B., Ribatti D. (2010). Aquaporins in tumor growth and angiogenesis. Cancer Lett..

[12] Papadopoulos M., Saadoun S., Verkman A. (2008). Aquaporins and cell migration. Pflügers Arch. Eur. J. Physiol..

[13] Tomita Y., Dorward H., Yool A.J., Smith E., Townsend A.R., Price T.J., Hardingham J.E. (2017). Role of aquaporin 1 signalling in cancer development and progression. Int. J. Mol. Sci..

[14] Jeong S.M., Lee J.-H., Kim J.-H., Lee B.-H., Yoon I.-S., Lee J.-H., Kim D.-H., Rhim H., Kim Y., Nah S.-Y. (2004). Stereospecificity of Ginsenoside Rg 3 Action on Ion Channels. Mol. Cells.

[15] Kim J.-H., Lee J.-H., Jeong S.M., Lee B.-H., Yoon I.-S., Lee J.-H., Choi S.-H., Kim D.-H., Park T.-K., Kim B.-K. (2006). Stereospecific effects of ginsenoside Rg3 epimers on swine coronary artery contractions. Biol. Pharm. Bull..

[16] Wei X., Su F., Su X., Hu T., Hu S. (2012). Stereospecific antioxidant effects of ginsenoside Rg3 on oxidative stress induced by cyclophosphamide in mice. Fitoterapia.

[17] Wei X., Chen J., Su F., Su X., Hu T., Hu S. (2012). Stereospecificity of ginsenoside Rg3 in promotion of the immune response to ovalbumin in mice. Int. Immunol..

[18] Wu R., Ru Q., Chen L., Ma B., Li C. (2014). Stereospecificity of Ginsenoside Rg3 in the promotion of cellular immunity in hepatoma H22-bearing mice. J. Food Sci..

[19] Kim Y.-J., Choi W.-I., Jeon B.-N., Choi K.-C., Kim K., Kim T.-J., Ham J., Jang H.J., Kang K.S., Ko H. (2014). Stereospecific effects of ginsenoside 20-Rg3 inhibits TGF-β1-induced epithelial–mesenchymal transition and suppresses lung cancer migration, invasion and anoikis resistance. Toxicology.

[20] Myers R.H., Montgomery D.C., Anderson-Cook C.M. (2016). Response Surface Methodology: Process and Product Optimization Using Designed Experiments.

[21] Razura-Carmona F.F., Pérez-Larios A., González-Silva N., Herrera-Martínez M., Medina-Torres L., Sáyago-Ayerdi S.G., Sánchez-Burgos J.A. (2019). Mangiferin-loaded polymeric nanoparticles: Optical characterization, effect of anti-topoisomerase I., and cytotoxicity. Cancers.

[22] Liou J.-Y., Tsou M.-Y., Ting C.-K. (2015). Response surface models in the field of anesthesia: A crash course. Acta Anaesthesiol. Taiwanica.

[23] Zafar S., Akhter S., Ahmad I., Hafeez Z., Rizvi M.M.A., Jain G.K., Ahmad F.J. (2020). Improved chemotherapeutic efficacy against resistant human breast cancer cells with co-delivery of Docetaxel and Thymoquinone by Chitosan grafted lipid nanocapsules: Formulation optimization, in vitro and in vivo studies. Colloids Surf. B Biointerfaces.

[24] Lee J.J., Kong M., Ayers G.D., Lotan R. (2007). Interaction index and different methods for determining drug interaction in combination therapy. J. Biopharm. Stat..

[25] Malfettone A., Silvestris N., Paradiso A., Mattioli E., Simone G., Mangia A. (2012). Overexpression of nuclear NHERF1 in advanced colorectal cancer: Association with hypoxic microenvironment and tumor invasive phenotype. Exp. Mol. Pathol..

[26] Smith E., Palethorpe H., Tomita Y., Pei J., Townsend A., Price T., Young J., Yool A., Hardingham J. (2018). The purified extract from the medicinal plant bacopa monnieri, bacopaside II, inhibits growth of colon cancer cells in vitro by inducing cell cycle arrest and apoptosis. Cells.

[27] Tomita Y., Palethorpe H.M., Smith E., Nakhjavani M., Townsend A.R., Price T.J., Yool A.J., Hardingham J.E. (2019). Bumetanide-derived aquaporin 1 inhibitors, AqB013 and AqB050 inhibit tube formation of endothelial cells through induction of apoptosis and impaired migration in vitro. Int. J. Mol. Sci..

[28] Palethorpe H., Tomita Y., Smith E., Pei J., Townsend A., Price T., Young J., Yool A., Hardingham J. (2018). The aquaporin 1 inhibitor bacopaside II reduces endothelial cell migration and tubulogenesis and induces apoptosis. Int. J. Mol. Sci..

[29] Jiao Q., Bi L., Ren Y., Song S., Wang Q., Wang Y.-s. (2018). Advances in studies of tyrosine kinase inhibitors and their acquired resistance. Mol. Cancer.

[30] Thieltges K.M., Avramovic D., Piscitelli C.L., Markovic-Mueller S., Binz H.K., Ballmer-Hofer K. (2018). Characterization of a drug-targetable allosteric site regulating vascular endothelial growth factor signaling. Angiogenesis.

[31] Brozzo M.S., Bjelić S., Kisko K., Schleier T., Leppänen V.-M., Alitalo K., Winkler F.K., Ballmer-Hofer K. (2012). Thermodynamic and structural description of allosterically regulated VEGFR-2 dimerization. Blood.

[32] Keung M.-H., Chan L.-S., Kwok H.-H., Wong R.N.-S., Yue P.Y.-K. (2016). Role of microRNA-520h in 20 (R)-ginsenoside-Rg3-mediated angiosuppression. J. Ginseng Res..

[33] Li J.-P., Zhao F.-L., Yuan Y., Sun T.-T., Zhu L., Zhang W.-Y., Liu M.-X. (2017). Studies on anti-angiogenesis of ginsenoside structure modification HRG in vitro. Biochem. Biophys. Res. Commun..

[34] Wenthur C.J., Gentry P.R., Mathews T.P., Lindsley C.W. (2014). Drugs for allosteric sites on receptors. Annu. Rev. Pharmacol. Toxicol..

[35] Grover A.K. (2013). Use of allosteric targets in the discovery of safer drugs. Med Princ. Pract..

[36] Muz B., de la Puente P., Azab F., Azab A.K. (2015). The role of hypoxia in cancer progression, angiogenesis, metastasis, and resistance to therapy. Hypoxia.

[37] Walsh J.C., Lebedev A., Aten E., Madsen K., Marciano L., Kolb H.C. (2014). The clinical importance of assessing tumor hypoxia: Relationship of tumor hypoxia to prognosis and therapeutic opportunities. Antioxid. Redox Signal..

[38] Luo D., Liu H., Lin D., Lian K., Ren H. (2019). The clinicopathologic and prognostic value of hypoxia-inducible factor-2α in cancer patients: A systematic review and meta-analysis. Cancer Epidemiol. Prev. Biomark..

[39] Ma Q., Reiter R.J., Chen Y. (2020). Role of melatonin in controlling angiogenesis under physiological and pathological conditions. Angiogenesis.

[40] Ramjiawan R.R., Griffioen A.W., Duda D.G. (2017). Anti-angiogenesis for cancer revisited: Is there a role for combinations with immunotherapy?. Angiogenesis.

[41] Carpini J.D., Karam A.K., Montgomery L. (2010). Vascular endothelial growth factor and its relationship to the prognosis and treatment of breast, ovarian, and cervical cancer. Angiogenesis.

[42] Weigand M., Hantel P., Kreienberg R., Waltenberger J. (2005). Autocrine vascular endothelial growth factor signalling in breast cancer. Evidence from cell lines and primary breast cancer cultures in vitro. Angiogenesis.

[43] Masłowska K., Halik P.K., Tymecka D., Misicka A., Gniazdowska E. (2021). The Role of VEGF receptors as molecular target in nuclear medicine for cancer diagnosis and combination therapy. Cancers.

[44] Tian T., Li X., Zhang J. (2019). mTOR signaling in cancer and mTOR inhibitors in solid tumor targeting therapy. Int. J. Mol. Sci..

[45] Chen M.C., Hsu W.L., Chang W.L., Chou T.C. (2017). Antiangiogenic activity of phthalides-enriched Angelica Sinensis extract by suppressing WSB-1/pVHL/HIF-1α/VEGF signaling in bladder cancer. Sci. Rep..

[46] Karar J., Maity A. (2011). PI3K/AKT/mTOR pathway in angiogenesis. Front. Mol. Neurosci..

[47] Guba M., von Breitenbuch P., Steinbauer M., Koehl G., Flegel S., Hornung M., Bruns C.J., Zuelke C., Farkas S., Anthuber M. (2002). Rapamycin inhibits primary and metastatic tumor growth by antiangiogenesis: Involvement of vascular endothelial growth factor. Nat. Med..

[48] Mi C., Ma J., Wang K.S., Zuo H.X., Wang Z., Li M.Y., Piao L.X., Xu G.H., Li X., Quan Z.S. (2017). Imperatorin suppresses proliferation and angiogenesis of human colon cancer cell by targeting HIF-1α via the mTOR/p70S6K/4E-BP1 and MAPK pathways. J. Ethnopharmacol..

[49] Saraswati S., Kumar S., Alhaider A.A. (2013). α-santalol inhibits the angiogenesis and growth of human prostate tumor growth by targeting vascular endothelial growth factor receptor 2-mediated AKT/mTOR/P70S6K signaling pathway. Mol. Cancer.

[50] Dodd K.M., Yang J., Shen M.H., Sampson J.R., Tee A.R. (2015). mTORC1 drives HIF-1α and VEGF-A signalling via multiple mechanisms involving 4E-BP1, S6K1 and STAT3. Oncogene.

[51] Herbert S.P., Costa G. (2019). Sending messages in moving cells: mRNA localization and the regulation of cell migration. Essays. Biochem..

[52] Willett M., Brocard M., Davide A., Morley S.J. (2011). Translation initiation factors and active sites of protein synthesis co-localize at the leading edge of migrating fibroblasts. Biochem. J..

[53] Li W., Petrimpol M., Molle K.D., Hall M.N., Battegay E.J., Humar R. (2007). Hypoxia-induced endothelial proliferation requires both mTORC1 and mTORC2. Circ. Res..

[54] Stein J., Milewski W.M., Hara M., Steiner D.F., Dey A. (2011). GSK-3 inactivation or depletion promotes β-cell replication via down regulation of the CDK inhibitor, p27 (Kip1). Islets.

[55] Hermida M.A., Kumar J.D., Leslie N.R. (2017). GSK3 and its interactions with the PI3K/AKT/mTOR signalling network. Adv. Biol. Regul..

[56] Guri Y., Colombi M., Dazert E., Hindupur S.K., Roszik J., Moes S., Jenoe P., Heim M.H., Riezman I., Riezman H. (2017). mTORC2 promotes tumorigenesis via lipid synthesis. Cancer Cell.

[57] Lamanuzzi A., Saltarella I., Desantis V., Frassanito M.A., Leone P., Racanelli V., Nico B., Ribatti D., Ditonno P., Prete M. (2018). Inhibition of mTOR complex 2 restrains tumor angiogenesis in multiple myeloma. Oncotarget.

[58] De Ieso M.L., Yool A.J. (2018). Mechanisms of aquaporin-facilitated cancer invasion and metastasis. Front. Chem..

[59] Nicchia G.P., Stigliano C., Sparaneo A., Rossi A., Frigeri A., Svelto M. (2013). Inhibition of aquaporin-1 dependent angiogenesis impairs tumour growth in a mouse model of melanoma. J. Mol. Med..

[60] Kaneko K., Yagui K., Tanaka A., Yoshihara K., Ishikawa K., Takahashi K., Bujo H., Sakurai K., Saito Y. (2008). Aquaporin 1 is required for hypoxia-inducible angiogenesis in human retinal vascular endothelial cells. Microvasc. Res..

[61] Saadoun S., Papadopoulos M.C., Hara-Chikuma M., Verkman A.S. (2005). Impairment of angiogenesis and cell migration by targeted aquaporin-1 gene disruption. Nature.

