# Supplementary material for: Anti-Angiogenic Properties of Ginsenoside Rg3 Epimers: In Vitro Assessment of Single and Combination Treatments
Source: Cancers (Basel). 2021 May 6;13(9):2223. doi: 10.3390/cancers13092223 (PMC8125638; doi:10.3390/cancers13092223)
Supplement: Supplementary file 1 [file cancers-13-02223-s001.zip › cancers-1159704-supplementary.pdf]

Supplementary data

Supplementary Table S1. Design table developed for the RSM model.

Run*	SRg3 (A)	RRg3 (B)
1	0	0
2	0	0
3	-1	1
4	0	1
5	-1	-1
6	0	0
7	1	-1
8	0	0
9	0	-1
10	-1	0
11	0	0
12	1	1
13	1	0

*See Table 1 for values corresponding to the bounds -1, 0 and 1.

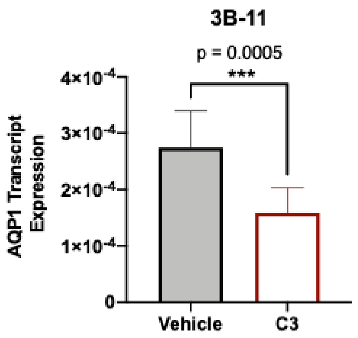

Supplementary Figure S1. Quantitative PCR for transcript expression of AQP1 in murine 3B-11 cell line following exposure with C3 for 3 days. PureLink RNA mini kit (Life Technologies, Grand Island, NY, USA) was used to extract RNA and 20 ng RNA was used for reverse transcription using iScript cDNA Synthesis Kit (Bio-Rad Laboratories, Hercules, CA, USA). The duplex TaqMan Gene Expression Assays for aquaporin-1 (AQP1; Mm01326465_g1; Applied Biosystems, Foster City, CA, USA) was used for the mouse endothelial cell line with *Eef2* (Eef2; Mm01171434_g1; Applied Biosystems, Foster City, CA, USA) as the reference gene. Reactions were performed in triplicate and analyzed as previously described [28].

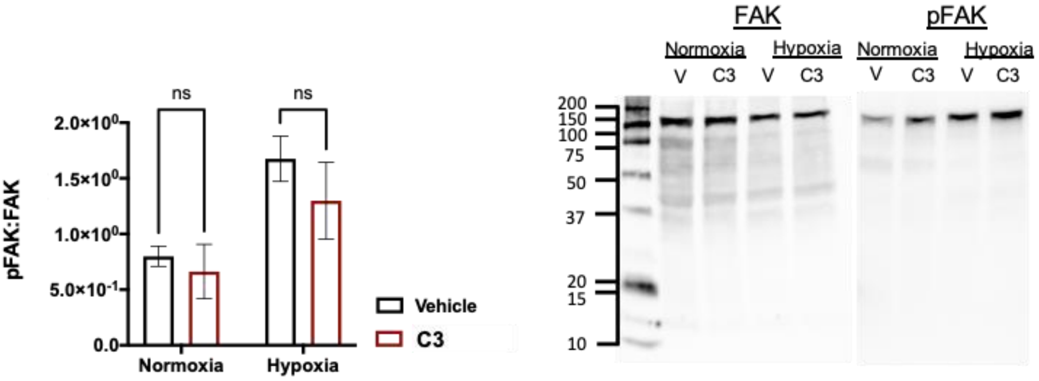

Supplementary Figure S2. Western blot analysis of activation of focal adhesion kinase (FAK) in HUVEC exposed to vehicle (V) or C3 (50 μM SRg3 + 25 μM RRg3), in normoxia or hypoxia conditions. Western blot was performed as previously described [28]. The antibodies used for immunostaining include anti-VEGF Receptor 2 antibody [EPRER16Y] (ab134191, Abcam, Cambridge, UK), anti-AQP1 antibody [EPR20325] (ab219055, Abcam, Cambridge, UK, 1:1000), anti-FAK antibody [EP6954] (ab40794, Abcam, Cambridge, UK, 1:1000), anti-phospho FAK antibody [EP2]60Y] phosphor Y397 (ab 81298, Abcam, Cambridge, UK, 1:1000). Goat anti-rabbit IgG H&L (HRP) (ab6721, Abcam, Cambridge, UK, 1:3000) was used as the secondary antibody. The experiments were repeated 3 times and the results are shown as mean ± SD.

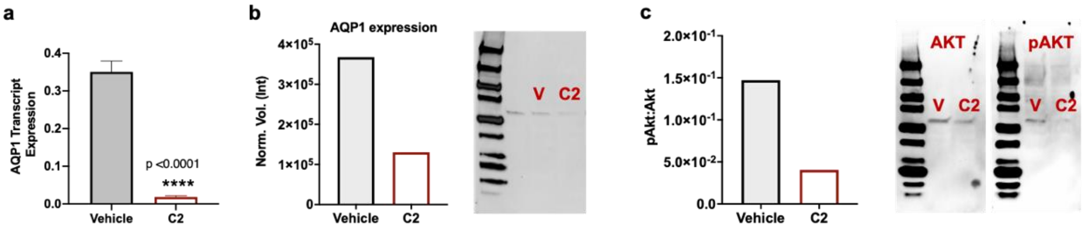

Supplementary Figure S3. HUVEC was exposed to vehicle (V) or C2 (25 μM SRg3 + 12.5 μM RRg3) for 3 days. (**a**) Quantitative PCR for transcript expression of AQP1 shown as mean ± SD of three experiments, (**b**) western blot protein expression of AQP1 and (**c**) activation of AKT in these cells. Anti-AQP1 antibody [EPR20325] (ab219055, Abcam, Cambridge, UK, 1:1000), anti-AKT antibody [E.32.10] (Invitrogen, Rockford, USA) and anti-phospho AKT antibody [104A282] (Invitrogen, Rockford, USA) were used.
